# Deep Learning Techniques for Spanish Sign Language Interpretation

**DOI:** 10.1155/2021/5532580

**Published:** 2021-06-14

**Authors:** Ester Martinez-Martin, Francisco Morillas-Espejo

**Affiliations:** Department of Computer Science and Artificial Intelligence, University of Alicante, E-03690 San Vicente del Raspeig, Alicante, Spain

## Abstract

Around 5% of the world population suffers from hearing impairment. One of its main barriers is communication with others since it could lead to their social exclusion and frustration. To overcome this issue, this paper presents a system to interpret the Spanish sign language alphabet which makes the communication possible in those cases, where it is necessary to sign proper nouns such as names, streets, or trademarks. For this, firstly, we have generated an image dataset of the signed 30 letters composing the Spanish alphabet. Then, given that there are static and in-motion letters, two different kinds of neural networks have been tested and compared: convolutional neural networks (CNNs) and recurrent neural networks (RNNs). A comparative analysis of the experimental results highlights the importance of the spatial dimension with respect to the temporal dimension in sign interpretation. So, CNNs obtain a much better accuracy, with 96.42% being the maximum value.

## 1. Introduction

Over 15% of the global population suffers from some form of disability and this rate is continuously growing (expected to double by 2050), according to the World Health Organization (WHO) [1]. Although there are several types of disability, all those people experience social and economic barriers when included in society. This is especially critical when it leads to communication exclusion, as it is the case of people with hearing loss (around 5% of the worldwide population [2]).

With the aim of overcoming this issue, a great variety of hearing aids have been developed. However, the use of these devices depends on the level of person's hearing loss. As a consequence, those aids are not adequate for hard-hearing and deaf community and, consequently, alternative ways to communicate are required. In this sense, there are a number of options such as sign language, lip reading, and using text; and their use will determine their form of communication. Nevertheless, although the sign language is learnt just as easily as hearing children learn spoken language when they are immersed in a signing community [3], it can also result in social isolation, since few people know this language. In addition, it is not an international language, which further complicates the communication process.

In this regard, some efforts can be found in the literature. One of the first attempts of recognising sign language without using *datagloves* is that presented by Starner and Pentland [4]. For that, one-colour images were processed by using the user's skin tone such that the user's hand shape, orientation, and trajectory were extracted. This data was the input to a Hidden Markov Model (HMM) for signed word recognition. In their experiments, a subset of the American Sign Language (ASL) was used. In particular, the considered words were the following:

- Pronouns: I, you, he we, you (plural), and they
- Verbs: want, like, lose, do not want, do not like, love, pack, hit, and loan
- Nouns: box, car, book, table, paper, pants, bicycle, bottle, can, wristwatch, umbrella, coat, pencil, shoes, food, magazine, fish, mouse, pill, and bowl
- Adjectives: red, brown, black, gray, and yellow

So, a total of 478 sentences together with extra information about with the context were used to train and test the system by getting an accuracy of 87.9% in training and 84.7% in test at a rate of 10 frames per second. With the purpose of improving their results, some features, such as the hand area, the length of the major axis of the first eigenvector, and the change in *x*- and *y*-positions of the hand, were added to help solve the ambiguity when the user's hands crossed. In this case, the accuracy increased up to 94.1% in training and 91.9% in test, while the best results were 81% and 74.5%, respectively, when no grammar context was used.

Along this line, Zaki and Shaheen [5] presented another ASL recogniser. In this case, the first step consisted in hand detection and tracking by means of a skin colour thresholding. Then, Principal Component Analysis (PCA) was used as a descriptor of hand configuration and orientation. Finally, HMM was in charge of sign classification. This approach got an accuracy of 89.1% on the *RWTH-BOSTON-50* dataset [6], which is composed of 50 American words signed by three people.

Another example is the work presented by Cooper et al. [7], where a subunit extraction is combined with HMM to get a German sign classification. Their experimental results show an accuracy of 85.1% on a 40-sign test set.

Going a step further, Pigou et al. [8] proposed Convolutional Neuronal Networks (CNNs) together with an Artificial Neural Network (ANN) to recognise 20 Italian gestures from the ChaLearn Looking at People 2014 dataset [9]. So, depth and gray images are input to a two-stream CNN of three layers each, where 2D convolutions and max pooling operations are combined. In this way, hand and upper body features are extracted. From those features, an ANN composed only of rectified linear units (ReLUs) with one hidden layer provides sign classification. This combination resulted in an accuracy of 91.70% on the validation set.

When entire sentences are considered, temporal segmentation and sentence synthesis are required. From this starting point, Fang et al. [10] presented DeepASL, a multilayer architecture for the translation of American Sign Language (ASL) sentences. So, 3D coordinates of the skeleton joints of fingers, palms, and forearms provided by Leap Motion are used to extract the key characteristics of ASL signs. These characteristics feed to a hierarchical bidirectional deep Recurrent Neural Network such that its output is translated by using Connectionist Temporal Classification (CTC). This system was evaluated by using the following words:

- Pronouns: who, I, you, what, we, my, your, and other
- Nouns: time, food, drink, mother, clothes, box, car, bicycle, book, shoes, year, boy, church, and family
- Verbs: want, do not want, like, help, finish, need, thank you, meet, live, can, and come
- Adjectives: big, small, hot, cold, blue, red, ray, black, green, white, old, with, without, nice, bad, sad, many, sorry, and few
- Adverbs: where, more, please, and but

These words were combined to generate 100 meaningful sentences for DeepASL evaluation. The results show a Top1-WER of 16.1 ± 3.7%, which means that, for a 4-word sentence, there is only an average of 0.64 words requiring either substitution, deletion, or insertion. In a similar way, Zhang et al. [11] proposed MyoSign, a deep learning-based system for ASL sentence recognition. As DeepASL, MyoSign uses a bidirectional Long Short-Term Memory (LSTM) followed by CTC. However, instead of images, EMG signals together with 3-axis accelerometer, gyroscope, and acceleration are the input for MyoSign. These signals are processed by a Convolutional Neural Network (CNN) that feeds into the bidirectional LSTM. An evaluation over 100 sentences resulted in an accuracy of 92.4%. Along this line, several approaches have been also proposed in the literature ([12–14]).

Despite the wide research in this area, each work has its own limitations in terms of cost, image preprocessing, and sign classification, as stated in [15]. In this paper, we analyse several deep learning architectures for Spanish Sign Language. In this sense, two different approaches are considered: spatial dimension and spatiotemporal analysis. In addition, we have created a dataset due to the lack of research on Spanish Sign Language.

### 1.1. Contributions

The specific contributions of this paper are as follows:

- Building our own dataset of over 8300 images belonging to the Spanish sign language alphabet
- Training and comparison of different CNNs as well as our own architecture for static Spanish sign letters interpretation
- Training and comparison of different Recurrent Neural Networks (RNNs) together with our proposed approach for Spanish sign language interpretation
- Analysis of performance in terms of Spanish sign language interpretation

## 2. LSE Dataset

As mentioned above, the sign language is not an international language. As a consequence, on the way to design a sign language recogniser and interpreter, it is necessary to create a dataset for each language with the signs to be learnt, except for the American Sign Language that has some public datasets [16, 17].

**Figure 1:**
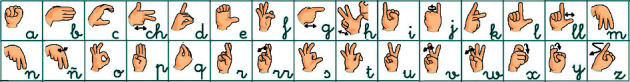
Spanish alphabet in sign language.

This lack of available data led to building our own dataset. For that, ten people (eight men and two women) were recorded while signing the Spanish sign language alphabet as depicted in Figure 1. As shown, a total of 18 letters are represented by a static gesture, whereas 12 letters require motion to be signed. This fact resulted in considering different deep learning techniques to properly recognise and interpret each letter.

An issue to be considered is that each person signifies by using their active or dominant hand. So, a right-handed person signifies with the right hand and, in fact, their main signed area is located between the right shoulder and the centre of the chest unlike left-handers. For this reason, all the subjects were required to signify all the letters during several seconds (between 10 and 20 seconds) firstly by using their dominant hand and then by their other hand. These recordings resulted in 8300 samples of hand gestures. More precisely, Table 1 shows the image dataset distribution among the Spanish alphabet letters.

**Figure 2:**
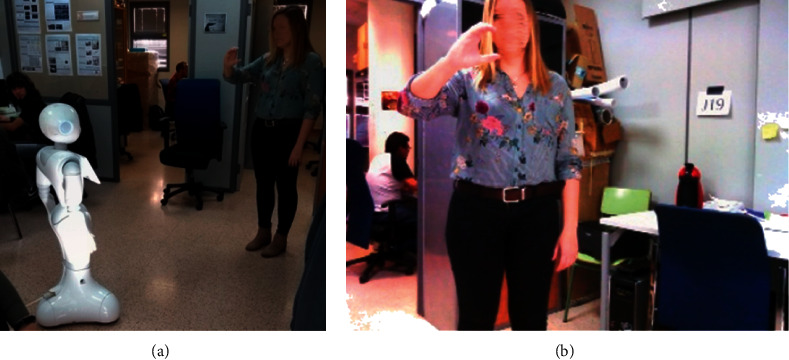
Sample of image capture. (a) Pepper capturing images while person signing. (b) Image captured by Pepper for the dataset.

The whole dataset was recorded using the RGB camera located in the head of a SoftBank Robotics Pepper robot [18]. This camera provides a resolution up to 2560 × 1920 at 1 frame per second. However, in our case, a 640 × 480 resolution at 30 frames per second was used. So, the *signers* were located in front of the robot at a distance between 1.5 and 2.5 metres from the robot, as shown in Figure 2, such that the robot was recording the images composing our dataset.

Although, as we can see in Table 1, there is a little imbalance in the samples of the dataset, it does not affect in an important way to the recognition. That is because the imbalanced letters (d, n, ñ, and t) differ in the spatial dimension from the rest, meaning that there is no problem for the system to recognise, for example, letter d from the rest of the alphabet even having less data for training. On the other hand, some letters such as a and e have more samples than the rest; that is because those two letters may get confused between them so a bit more samples help to reduce that confusion.

**Figure 3:**
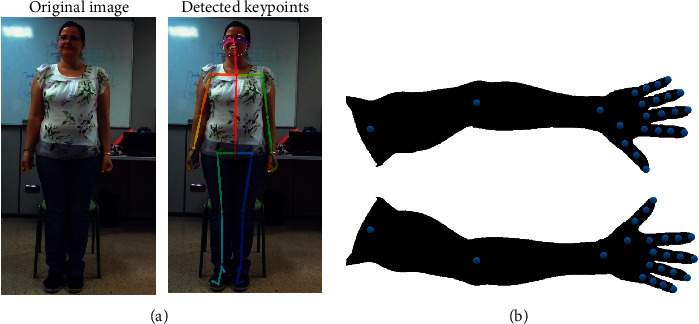
Openpose keypoints. (a) All the 135 Openpose keypoints linked by lines. (b) Arm and hand joints provided by Openpose.

**Figure 4:**
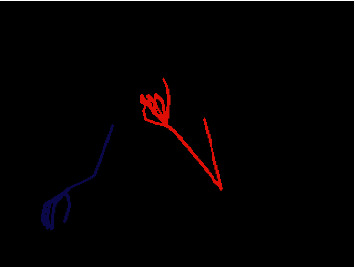
Image dataset sample with colour-coded sign elements.

Since the designed system must pay attention to signer's hands and arms, the RGB images were processed to extract those features. With that purpose, Openpose [19, 20] was used. This open-source library detects anatomical keypoints on single images in real time through a multistage Convolutional Neural Network (CNN). So, its 2D keypoint detector outputs 135 keypoints per person: 25 keypoints correspond to the human skeleton; 40 keypoints represent the hands, while 70 keypoints are used for the face (see Figure 3(a)). According to the signing specifications, 46 keypoints are used in this work as illustrated in Figure 3(b). These keypoints are connected by lines and drawn on 240 × 320 colour images such that the left side is coded in red, while the right side is blue-coloured as shown in Figure 4. All these generated 240 × 320 × 3 images are the ones composing our dataset.

## 3. Sign Language Interpretation

Sign language recognition can be described as an image classification task, where an input image will output its corresponding meaning. In this sense, current approaches make essential use neural network techniques. In particular, CNNs have been proven to be very successful in image recognition. However, a challenging issue is to find a way to distinguish two letters whose only difference is to be moved or not, like l and ll. As a solution, RNNs could be considered, since they are designed to take a series of inputs and provide an output based on temporal analysis and the learnt meaningful relationships between the input data. From this starting point, several architectures of both types are analysed and compared. Note that both types of architectures have been compared with the aim to study the influence of the spatial and temporal dimensions in the sign language interpretation.

### 3.1. Convolutional Neural Network (CNN) Approaches

The issue of sign interpretation can be defined as a visual classification task. In this context, a popular approach is Convolutional Neural Networks (CNNs). This approach is inspired in the human visual cortex. The underlying idea is to use multiple layers that perform discrete convolutions together with activation functions and other operations like pooling to get a classification tag. In the following sections, the CNNs used in this paper are presented.

#### 3.1.1. VGG

**Figure 5:**
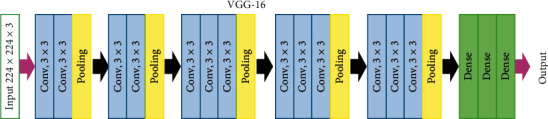
VGG-16 architecture [21].

VGG is a deep CNN architecture introduced by Simonyan and Zisserman [21]. As illustrated in Figure 5, this well-known architecture is basically composed of four types of layers: convolutional, max pooling, activation, and fully connected. In particular, this architecture addresses an important aspect of CNNs: depth. Therefore, it uses very small receptive field (3 × 3 with a stride of 1 (the smallest size to capture the notion of left/right, up/down, centre)). These small-size convolution filters allow VGG to have a large number of layers, which leads to an improved performance. Although this model supports up to 19 layers, the architecture with 16 layers (VGG-16) is used in this paper.

#### 3.1.2. Inception V3

Inception V3 [22] is the third version in the Inception network family. The first version (Inception V1) had 7 million parameters and was presented as GoogLeNet in 2015. It introduced the Inception module, where the input simultaneously goes through 1 × 1, 3 × 3, and 5 × 5 convolutions to look at both cross-channel correlations and spatial correlations. The introduction of batch normalization together with other architecture refinements led to the second version (Inception V2). On its behalf, Inception V3 included additional factorization ideas in convolution layers to reduce the dimensionality and the overfitting problem. This fact resulted in a reduction of a third of the parameters. In addition, an efficient grid size reduction was also introduced, reducing the computational cost while keeping the efficiency. The fourth and last version, Inception V4 (also called Inception-ResNet), added residual connection like ResNet's own.

**Figure 6:**
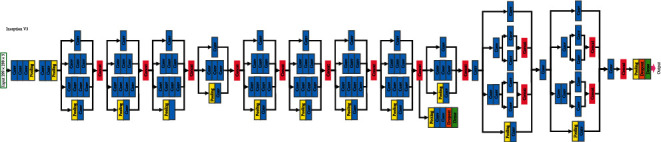
Inception V3 architecture [22].

In particular, in this paper, Inception V3 is used. The layout of this architecture can be seen in Figure 6.

#### 3.1.3. Xception

Xception [23] is a neural network inspired by Inception. However, unlike Inception, Xception is based on the assumption that cross-channel correlations and spatial correlations in the feature maps are entirely decoupled. So, the Inception modules are replaced for depthwise separable convolutions (a point-wise convolution (1 × 1 convolution) followed by a depthwise convolution (a channel-wise nxn spatial convolution)). Broadly speaking, Xception can be defined as a linear stack of depthwise separable convolution layers with residual connections like ResNet's own.

#### 3.1.4. ResNet

**Figure 7:**
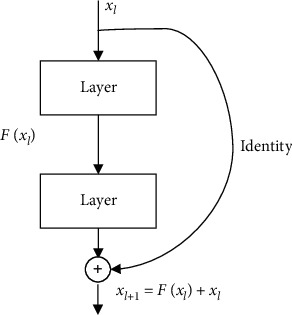
ResNet residual block.

Residual Network (ResNet) [2, 24] is one of the most popular deep neural networks for image classification. In contrast to prior networks that increase the network depth to get a higher performance, ResNet introduced the concept of identity connection between layers by resulting in residual blocks like that illustrated in Figure 7. Basically, these connections skip one or more layers to obtain identity maps. Given that these connections do not add neither extra parameters nor computational complexity, they avoid an increase in the model training error when getting deeper. As previously, a family of ResNet models have been implemented scaling up from 18 to 200 layers.

In this paper, 50-layer ResNet is used. This network can be summarised as follows: an initial convolution and max pooling perform input downsampling. After that, four stages combining convolutional layers with identity connections are performed such that the channel width is doubled in each stage, while the size of the input is reduced to half. Finally, an average pooling layer followed by a fully connected one provides a classification tag.

#### 3.1.5. EfficientNet

EfficientNet [6] arose from the search of a new way to scale CNN models such that a better accuracy and efficiency were achieved. So, unlike the conventional practice to arbitrarily increase the CNN depth (number of layers), width, or input image resolution, a compound scaling method to uniformly scale all of them is proposed. For that, a set of fixed scaling coefficients are obtained from the relationship between the three dimensions. Then, these coefficients are used to scale the baseline model up to the desired size or computational budget.

**Figure 8:**
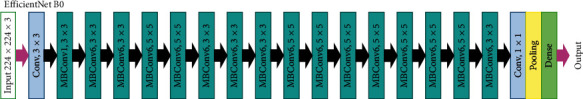
EfficientNet B0 baseline architecture.

From this starting point, seven models have been developed based on the baseline CNN model shown in Figure 8. These models were called EfficientNet-Bi, where *i* goes from 0 (the baseline model) to 7, the model with higher depth, width, and input resolution. In this paper, the intermediate model has been used, that is, EfficientNetB3.

#### 3.1.6. Our Own Architectures

**Figure 9:**
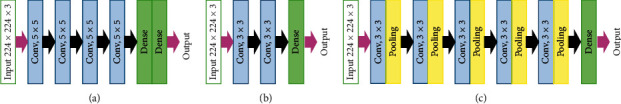
Our own CNN architectures. (a) LSE-CNN1. (b) LSE-CNN2. (c) LSE-CNN3.

In addition to the previous state-of-the-art CNNs, we also propose three different architectures to learn and interpret the sign language. So, as depicted in Figure 9, a combination of several kinds of layers (e.g., convolution, 2D convolution, fully connected, pooling, etc.) has been used as follows:

  LSE-CNN1: this architecture is composed of 6 layers. So, visual features of the 224 × 224 × 3 input images are extracted by means of the first four convolutional layers. All these layers use 5 × 5 kernels, although the number of filters changes by being 20 for the first two layers and 50 for the two last ones. The feature array is then flattened and processed by two fully connected layers such that the first one counts with 200 units, while the second one uses the total number of classes.
  LSE-CNN2: this is a more simplified architecture where a 2D convolutional layer with 64 filters feeds another 2D convolutional layer with 32 filters (both of them with a kernel size of 3 × 3). After flattening, a fully connected layer is applied for classification.
  LSE-CNN3: in this case, not only is the extraction of visual features based on convolutional operations, but also pooling layers are used. In particular, as shown in Figure 9, each convolutional layer is followed by a maxpooling layer such that the feature maps are downsampled by summarising the most activated presence of a visual feature in each step. In addition, the number of filters corresponding to the convolutional layers is doubling in each step going from 16 to 256. Again, a fully connected layer is applied after flattening for image classification.

### 3.2. Recurrent Neural Network (RNN) Approaches

Recurrent neural network (RNN) is a type of neural network, where inputs and outputs are temporally connected by means of loops. This fact makes them applicable to tasks where time and sequence must be taken into account like hand-writing recognition or speech recognition.

**Figure 10:**
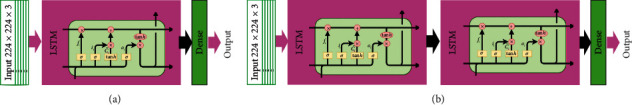
Our own RNN architectures. (a) LSE-RNN1. (b) LSE-RNN2.

One of the most well-known and powerful RNNs is the Long Short-Term Memory (LSTM) network. These networks were proposed by [25] with the aim to remember information while avoiding the vanishing gradient problem. For that, it uses a cell state regulated by gates. Given its efficiency including the temporal dimensionality, LSTMs were also used in our study. So, as illustrated in Figure 10, the first proposed architecture (LSE-RNN1) uses an LSTM layer with 32 units followed by a dense layer to interpret the sign language. On the contrary, the LSE-RNN2 uses two consecutive LSTM layers, each with 32 units and whose result inputs to a dense layer. That is, the difference between the two proposed RNN architectures is the number of used LSTM units. Thus, both architectures get as input a sequence of 224 × 224 × 3 images. Note that the length of this input image sequence is variable, since it depends on the performed sign and the signing person. In particular, in our case, this length varies between 2 and 10 images.

## 4. Experimental Results

As previously mentioned, two types of signs can be distinguished: those requiring motion and those static. So, firstly, the interpretation of sign language is analysed by only considering the spatial dimension and, consequently, the static signs are used. Then, the analysis is extended by taking into account both spatial and temporal dimensions. In this case, all the alphabet letters are considered.

### 4.1. First Experiment: Static Signs

**Figure 11:**
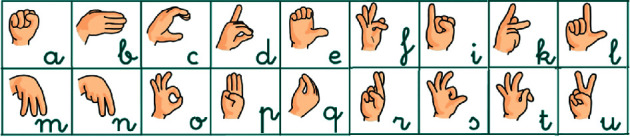
Spanish static signs considered for the first experiment.

The first experiment consists in the evaluation of the performance when static signs are considered. As illustrated in Figure 11, there are 18 static signs in the Spanish sign language. So, the key issue is the spatial distribution of the hand fingers signing the different letters. Note that there are some letters that can lead to confusion. In particular, the letters f, s, and t could be difficult to distinguish, since the difference in the position of the ring finger is subtle.

Given that the analysed architectures work with different input sizes, all of them were adapted to start with a 224 × 224 × 3 image size. Therefore, the first step was to resize all the 320 × 240 dataset colour images to the required dimensions. Then, the data was divided into three subsets as follows: 70% of the samples were used for training, 15% were used for test during the training, and the remaining 15% were used for performance validation. Note that most of the samples chosen for validation belong to a person whose images were not used for neither training nor test. Next, the several architectures were trained from scratch during 50 epochs on an Intel(R) Core(TM) i7-8700 CPU at 3.20 GHz with a GeForce RTX 2080 Ti. The accuracy obtained on the validation set together with the test set (that used during training) is summarised in Table 2. As can be observed, except for the VGG-16 architecture, all the accuracies are greater than 85% in both validation and test, getting a maximum validation accuracy of 96.16% (3.84% error rate) for the best model, that is, Efficient-NetB3. Note that the test result is higher than the validation result because all the validation set was not previously seen.

**Figure 12:**
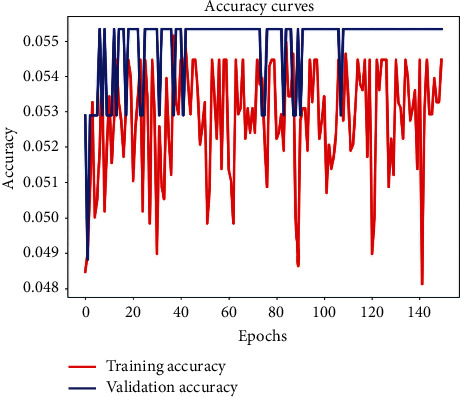
VGG16 accuracy curve for 150 epochs.

It is worth noting that, in the case of VGG-16 architecture, it seems to provide random results. Given the high number of parameters to be adjusted (i.e., 134 387 551), a longer training was performed. As illustrated in Figure 12, the VGG-16 accuracy curve is fluctuating. This fact is a consequence of overfitting. So, VGG-16 architecture is not able to properly extract the desired information from the provided data.

**Figure 13:**
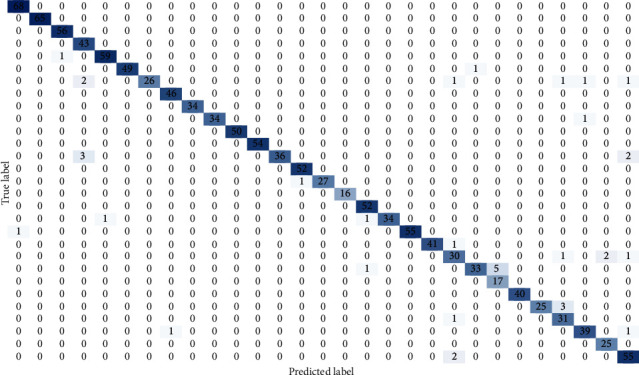
EfficientNetB3 confusion matrix on the validation test when static signs are considered.

An analysis of the results shows that, as expected, the most confused letter is s, since it can be mainly confounded with f and t, as illustrated in the confusion matrix of the best model in Figure 13.

Another issue to be taken into account is the computational cost in processing an image and interpreting the sign. In this regard, Table 3 shows the times in seconds for each architecture. Although the best model provides a good performance with only 0.04339 seconds per frame, the best processing time is obtained with our proposed architecture LSE-RNN1. However, this architecture gets much lower accuracy falling down up to 89.24% which implies a loss of almost 7%. On the contrary, another of our proposed architectures, LSE-CNN3, only requires 0.00199 seconds per frame. In this case, this architecture has also been shown to be efficient in the sign language recognition, since a validation accuracy of 94.37% is achieved (only an accuracy of 1.79% is lost). (Figure 13)

### 4.2. Second Experiment: The Whole Alphabet

Once the static sign recognition is analysed, the next experiment involves all the Spanish Sign Language alphabet. In this case, there are some letters requiring motion to be performed which means that temporal dimension must be considered. As previously, the data was resized to 224 × 224 × 3, divided into training, validation, and test (70%, 15%, and 15%, respectively) and input to the different architectures. In this case, the number of epochs was increased to 100 with the aim to properly learn the temporal pattern. In addition, a variable sequence size was established for the RNNs architectures, since each sign requires a different number of frames to be performed. Note that the static signs were grouped according to the person signing, the used hand, and the changes in position. This experiment leads to the results illustrated in Table 4. As previously, except for VGG-16 architecture, all the validation accuracies are above 85%. In this case, the best model is ResNet50 with a validation accuracy of 96.42% (3.58% error rate), although EfficientNetB3 gets the second best result (like Xception) with a validation accuracy of 95.77% (only a loss of 0.65%). The precision lost for the case of LSE-CNN3 increases up to 3.58%. On the contrary, the RNNs present a low validation accuracy without reaching the 87%. The main reason for that lies in the fact that spatial dimension has a much greater weight in the interpretation task because the position of fingers is quite different from one letter to another. The only exception is four pairs of letters whose only difference is movement: l-ll, n-ñ, r-rr, and u-v. This displacement can lead to a misclassification in some samples. Nevertheless, in the view of results, it seems that CNNs learn the position displacement to properly distinguish between these pairs of letters. Moreover, a feature of the signing process is that the participants changed the starting position depending on the motion requirement (or lack thereof). This is not the case of the triplet f-s-t that still confuses the networks.

## 5. Conclusions

In this paper, we have presented a new Spanish Sign alphabet dataset composed of over 8300 colour images which was obtained by representing the person's upper limbs. Then, we have discussed several ways to recognise the Spanish Sign alphabet distinguishing between two types of signs: those requiring movement and those static. This fact implies the analysis of both spatial and temporal dimensions. For that reason, two different types of architectures have been studied: CNNs, focused on the spatial dimension, and RNNs, aimed to process temporal sequences. So, a total of 10 architectures have been used in this paper such that five of them are well-known state-of the-art approaches, while the other five correspond to our own proposals.

The experimental results revealed that spatial dimension has much greater weight than temporal dimension in sign interpretation, since the RNNs got the lowest results. This is mainly due to the importance of the finger's position in front of the movement needed to sign, at least, in the Spanish Sign Language alphabet. Thus, these results show the generalization capability of CNNs in spatiotemporal data that can contribute to the broader research field on automatic sign language recognition. However, it is important to consider that subtle differences in fingers' position can make CNN approaches fail, as brought to light by the triplet f-s-t.

In addition, a temporal analysis was also performed. The models with the best accuracies take over 0.04 seconds per frame. A special attention is paid to one CNN architecture proposal, LSE-CNN3, which only takes 0.00199 seconds, while its accuracy is just reduced 1.79% for static signs and 3.58% for the whole alphabet with respect to the best model.

As future work, we plan to extend the dataset and the comparative study to include words and sentences aimed to completely cover the communication problem. In addition, the adaptation to other sign languages will also be analysed.

## Figures and Tables

**Table 1:** Data composition of our own dataset for the Spanish alphabet.

Letter	Left-hand sign	Right-hand sign	Total
a	227	227	454
b	222	222	444
c	192	192	384
ch	136	146	282
d	59	51	110
e	201	201	402
f	166	166	332
g	117	100	217
h	144	172	316
i	115	115	230
j	104	133	237
k	168	168	336
l	180	180	360
ll	139	138	277
m	173	173	346
n	90	90	180
ñ	61	53	114
o	175	175	350
p	122	122	244
q	184	189	373
r	152	152	304
rr	116	109	225
s	130	130	260
t	63	63	126
u	134	134	268
v	93	96	189
w	112	104	216
x	140	132	272
y	54	120	174
z	206	179	385

**Table 2:** Experimental results on static Spanish alphabet letters with 50 epochs.

Architecture	Accuracy (validation) (%)	Accuracy (test) (%)
VGG-16	8.51	8.51
Inception V3	93.87	95.87
Xception	90.99	92.37
ResNet50	95.99	**98.37**
EfficientNetB3	**96.16**	96.75
LSE-CNN1	87.36	86.61
LSE-CNN2	86.73	87.86
LSE-CNN3	**94.37**	**95.74**
LSE-RNN1	89.24	89.61
LSE-RNN2	87.23	88.86

Values in bold correspond to the best obtained results.

**Table 3:** Computational cost per image when running on an Intel(R) Core(TM) i7-8700 CPU at 3.20 GHz with GeForce RTX 2080 Ti.

Architecture	Time per frame (secs)
VGG-16	0.06389
Inception V3	0.01976
Xception	0.04341
ResNet50	0.03587
EfficientNetB3	0.04339
LSE-CNN1	0.03187
LSE-CNN2	0.00675
LSE-CNN3	**0.00199**
LSE-RNN1	**0.00043**
LSE-RNN2	0.03758

**Table 4:** Experimental results on the whole Spanish alphabet letters for 100 epochs.

Architecture	Accuracy (validation) (%)	Accuracy (test) (%)
VGG-16	5.53	5.53
Inception V3	94.79	95.93
Xception	95.77	**97.8**
ResNet50	**96.42**	97.31
EfficientNetB3	95.77	96.99
LSE-CNN1	86.17	85.76
LSE-CNN2	87.88	87.96
LSE-CNN3	**92.84**	**93.00**
LSE-RNN1	86.25	86.62
LSE-RNN2	86.74	86.74

Values in bold correspond to the best obtained results.

## Data Availability

The image data used to support the findings of this study are available from the corresponding author upon request.
